# Elemental Composition of Skeletal Muscle Fibres Studied with Synchrotron Radiation X-ray Fluorescence (SR-XRF)

**DOI:** 10.3390/ijms23147931

**Published:** 2022-07-19

**Authors:** Paula Kasprzyk, Paweł M. Wróbel, Joanna Dudała, Kalotina Geraki, Magdalena Szczerbowska-Boruchowska, Edyta Radwańska, Roger M. Krzyżewski, Dariusz Adamek, Marek Lankosz

**Affiliations:** 1 Faculty of Physics and Applied Computer Science, AGH University of Science and Technology, Al. Mickiewicz 30, 30-059 Krakow, Poland; pwrobel@agh.edu.pl (P.M.W.); joanna.dudala@fis.agh.edu.pl (J.D.); magdalena.boruchowska@fis.agh.edu.pl (M.S.-B.); 2 Diamond Light Source, Harwell Science and Innovation Campus, Didcot OX11 0DE, Oxfordshire, UK; tina.geraki@diamond.ac.uk; 3 Chair of Pathomorphology, Department of Neuropathology, Medical College, Jagiellonian University, Grzegórzecka 16 Str., 31-531 Krakow, Poland; earadwanska@tlen.pl (E.R.); mnadamek@cyf-kr.edu.pl (D.A.); 4 Department of Neurosurgery and Neurotraumatology, Medical College, Jagiellonian University, Jakubowskiego 2 Str., 30-688 Krakow, Poland; roger.krzyzewski@uj.edu.pl

**Keywords:** muscle disease, muscle fibres, myopathy, dystrophy, synchrotron radiation X-ray fluorescence (SR-XRF)

## Abstract

Diseases of the muscle tissue, particularly those disorders which result from the pathology of individual muscle cells, are often called myopathies. The diversity of the content of individual cells is of interest with regard to their role in both biochemical mechanisms and the structure of muscle tissue itself. These studies focus on the preliminary analysis of the differences that may occur between diseased tissues and tissues that have been recognised as a reference group. To do so, 13 samples of biopsied human muscle tissues were studied: 3 diagnosed as dystrophies, 6 as (non-dystrophic) myopathy and 4 regarded as references. From these sets of muscle biopsies, 135 completely measured muscle fibres were separated altogether, which were subjected to investigations using synchrotron radiation X-ray fluorescence (SR-XRF). Muscle fibres were analysed in terms of the composition of elements such as Br, Ca, Cl, Cr, Cu, Fe, K, Mn, P, S and Zn. The performed statistical tests indicate that all three groups (dystrophies—D; myopathies—M; references—R) show statistically significant differences in their elemental compositions, and the greatest impact, according to the multivariate discriminate analysis (MDA), comes from elements such as Ca, Cu, K, Cl and S.

## 1. Introduction

Pathological conditions that affect skeletal muscles, commonly termed myopathies, represent a broad spectrum of clinical and pathological changes. Some of them, such as muscle dystrophies, have a genetic background. Generally, they can be divided into two categories: primary and secondary. The first category encompasses a panoply of genetic disorders, including an important group collectively called dystrophies and other inherited myopathies; in the second category, one can especially distinguish disorders of the nervous system (e.g., leading to so-called denervation or neurogenic atrophy of muscle), toxic injury (including medication) and different metabolic, endocrine and immunological disorders. However, in many cases, the division into primary and secondary myopathies is difficult, unclear and even artificial—for instance, inflammatory myopathies that could be regarded as “primary”, with so-called inclusion body myositis (IBM) being an example—but in many myositides, there is evident overlap with essentially “non-muscular” conditions such as connective tissue diseases. Meanwhile, in many muscular dystrophies, not only skeletal muscles are affected, and the clinical picture of the disease includes disturbances of other organs, such as cataracts (in myotonic dystrophy); in turn, so-called mitochondrial myopathies may be involved. However, from the clinical point of view, it is obviously important to know whether the disease is essentially “muscular” or rather a “multi-organ” condition. Diseased muscle tissue can show numerable morphological (microscopic) changes, though the most typical (and essentially encountered in almost all “pathological” biopsies) is muscle fibre atrophy, which is represented by the diminishing of the muscle fibre cross-section dimension in a histological slide. As a result, being so unspecific as a common denominator of divergent muscle pathologies, its molecular mechanisms most probably are not the same in different types of myopathies.

This opens the possibility for investigation of the elemental and different biomolecular patterns of muscle atrophy and, in broader aspects, other pathological changes in diseased muscle.

The diagnosis of a muscle disease based on biopsy is, of course, not limited to investigation of the morphological changes observed under the microscope, but also involves several different methods of staining, including histochemical staining once and multiple immunohistochemical stainings with a broad spectrum of antibodies against different molecular constituents of muscle cells and cells that may “invade” muscle, such as lymphocytes, etc. It is worth noting that the interpretation of such multi-aspect pathology is very demanding and may be biased by some degree to subjectivity in assessment and conclusions, and the whole process of diagnosis is difficult; what is more, one also has to include the clinical picture of the diseases [1,2,3,4].

Since, as mentioned above, myopathies are diseases related to muscle fibres that lead to their decline and structural changes, their biomolecular and elemental composition may change as well. Those changes may therefore reflect the biochemical processes taking place in the fibres, so investigation of those changes may improve the understanding of the disease development at its early stages.

To the best of our knowledge, there is a lack of research related to measuring differences in the elemental composition of diseased human muscle tissue. Therefore, *for the first time, we* herein present the use of the synchrotron radiation X-ray fluorescence (SR-XRF) method to examine whether there are the differences in elemental composition between muscle fibres affected by disease and apparently healthy fibres—i.e., those that at least do not show microscopic features of pathology. The SR-XRF technique is a well-known gold-standard multi-elemental analytical method that enables the simultaneous tissue micro-imaging of chemical elements at trace concentrations (~mg/kg). A great advantage of the technique in muscle studies is its ability to simultaneously determine both chemical elements that play a key role in important processes, such as ion transport systems (Cl, K and Ca), energy metabolism (P and Fe) and enzymatic reactions (Fe, Cu, Zn and Mn), or are simply the components of biological macromolecules (P and S) as well as those whose importance for skeletal muscle function is less well understood (Cr and Br). Therefore, all the elements that could be determined using the SR-XRF technique were taken into account in the analysis [5,6,7].

## 2. Results and Discussion

**Figure 1:**
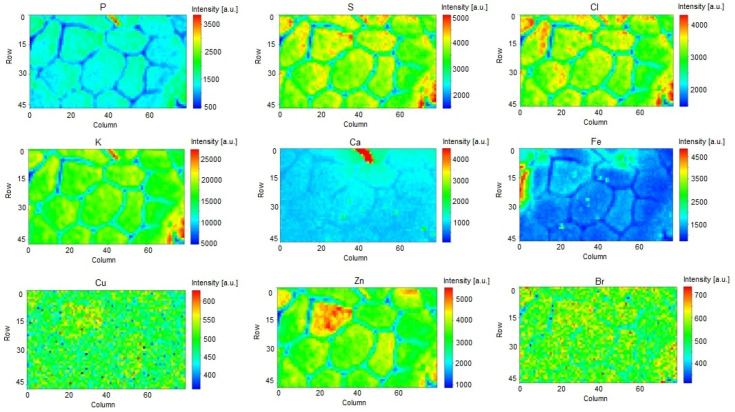
Element distribution maps for selected elements for a sample from the reference group.

The elemental intensity maps (Figure 1) show that the spatial distribution of elements in the muscle fibres is very heterogeneous, which can have a large impact on the performed analyses. For this reason, the fibres that were partially outside the scanning area were excluded from the statistical analysis. The overall dataset included 135 isolated and irregular fibres, for which 11 elements were analysed in each fibre. For the muscle fibres thus selected, the values of the normalised peak areas of each element were used in further statistical analysis. First, the data were evaluated using the Shapiro–Wilk test to check their agreement with the normal distribution. Based on the obtained results, it was found that the data did not show normal distribution. Therefore, the further statistical analysis was based on non-parametric tests.

**Figure 2:**
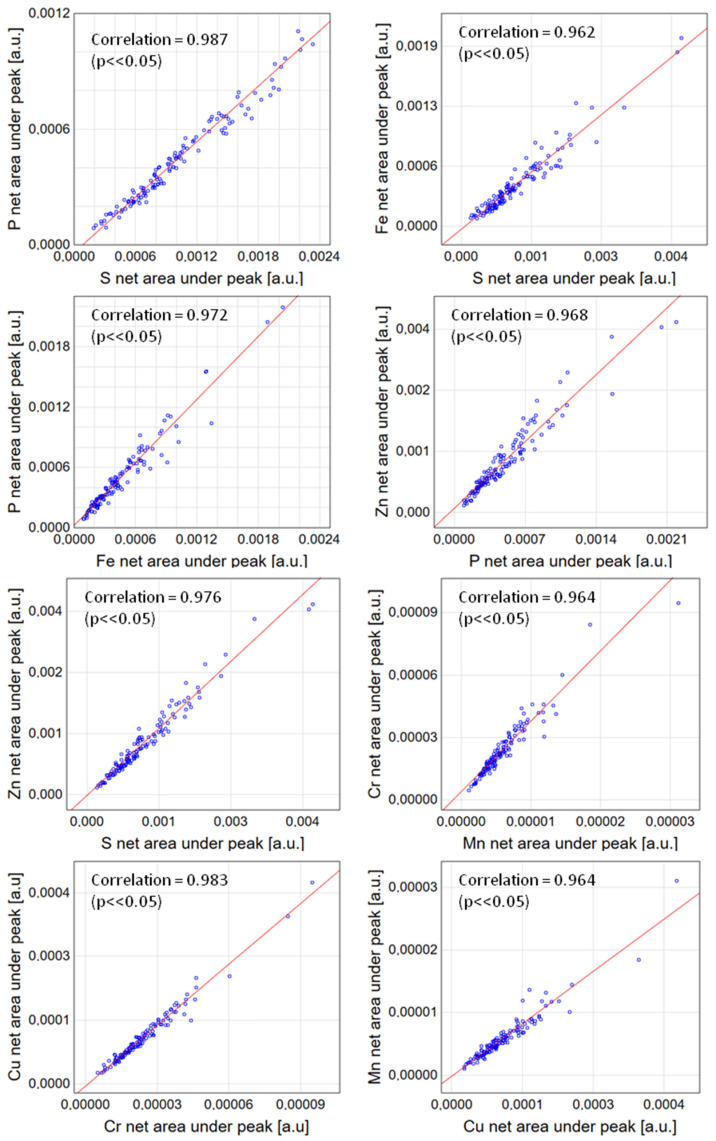
Correlation graphs for elements with the strongest dependence—correlation coefficient > 0.95.

For all analysed elements (without separation into types), the cross-correlation was checked using the Spearman correlation analysis (Figure 2). All the obtained correlation coefficients were considered statistically significant if the parameter *p* had a value < 0.05. A surprisingly large part of the elements showed a very strong linear relationship with a correlation coefficient value above 0.9. All the relationships turned out to be positive, indicating overlapping elevated concentrations. The strongest dependence (value above 0.95) was shown by paired elements, such as P and S, P and Fe, P and Zn, S and Fe, S and Zn, Mn and Cr, Cr and Cu and Cu and Mn.

**Figure 3:**
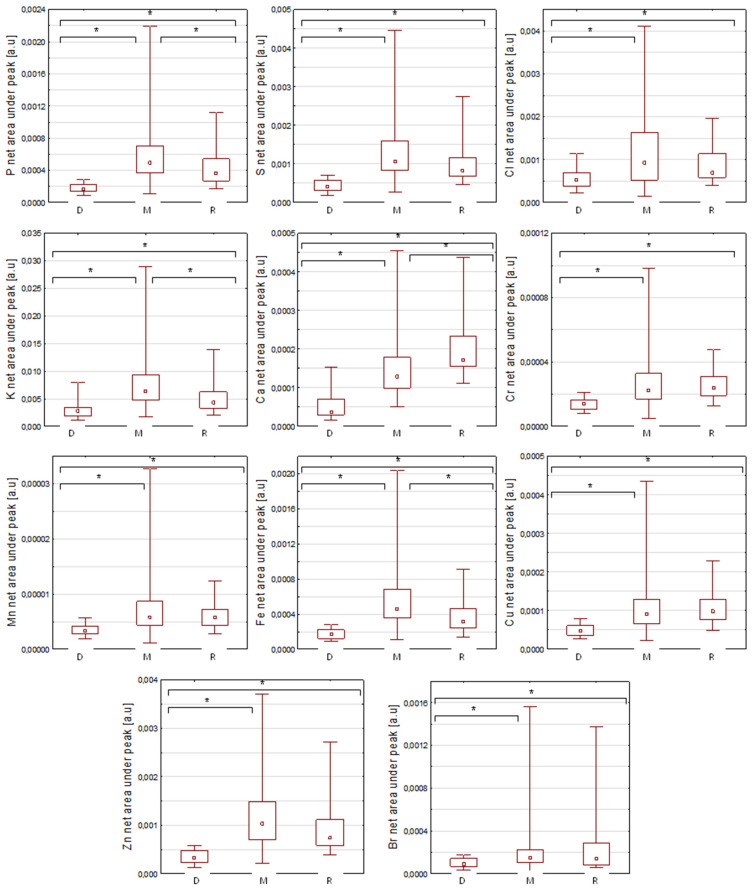
Box plots from the Kruskal–Wallis test for the individual elements (median, ±0.25–0.75% percentile and min–max values). Abbreviations: *—*p* < 0.05; D—dystrophies group; M—myopathy group; R—reference group.

The Kruskal–Wallis test was performed in order to check whether there are statistically significant differences between the groups for the analysed elements. In order to eliminate the outliers, the two-sided Tukey test [8] was used, with the change of the value into the mean. The values obtained as a result of the test were considered statistically significant when the *p* parameter obtained a value of 0.05 or less; for values above this number, the test result was considered statistically insignificant. The test results showed that for all elements, at least one group showed statistically significant differences. A post hoc test was performed in order to verify which groups show differences. For all groups, elements such as P, K, Ca and Fe were considered statistically significant in the differentiation of fibres. However, for the myopathy–reference pair elements, S, Cl, Cr, Mn, Cu, Zn and Br were considered statistically insignificant. The results of the intergroup comparison are presented for individual elements in Figure 3. For all the elements presented, it can be seen that the values of the normalised intensities of characteristic X-rays are much lower for the fibres from “dystrophic cases”. Here, it is worth remembering that dystrophies are muscle diseases that are inherited. The mutated genes associated with dystrophies are numerous, and the mode of inheritance may be either autosomal dominant, autosomal recessive or X-linked. Furthermore, the encoded proteins may be structural constituents of the cellular membrane of the myofiber (sarcolemma) or the constituents of the membrane that envelops the nuclei of muscle fibres (nucleolemma), or they may play some other particular functions. Mutation leads either to total or partial lack of expression of the encoded protein, or to its malfunction. The ultimate result of the mutation is therefore either structural or functional disintegration of muscle fibre, though the dynamics of the changes differ very much. The loss of elements investigated in this study in muscle fibres in dystrophies may suggest a sort of common pathway of pathological processes in dystrophies or (at least) simply underscore the level of cellular metabolic derangements which take place during disease. Each particular element plays a specific role in biomechanisms. Sulphur deficiency makes it difficult to absorb certain minerals. Chlorine deficiency causes muscle cramps and a weakening of muscle strength. Elements such as potassium and calcium are responsible for nerve conduction and muscle contraction processes; their deficiency may, therefore, cause problems with mobility. Iron is an essential component of myoglobin, which supplies oxygen to the muscles to produce energy. Copper deficiency causes muscle weakness and anaemia. Zinc, as an antioxidant that affects the development and regeneration of muscle tissue, also stabilises the protein structures from which fibres are made [9,10,11,12,13,14].

In order to find out which elements have the greatest influence on differentiation, a multivariate discriminate analysis (MDA) was performed for the analysed fibres. Log transformation [15] was used to normalise the data. From each group, 70% of the randomly selected fibres were used to create the model. Based on the partial Wilks’ lambda (Table 1), it was concluded that elements such as Ca, K, Cr and P made the greatest contribution to the separation of the measured fibre. S, Mn, Fe, Zn and Br were outside the discrimination model due to the overly high value of the *p* parameter (*p* > 0.05). The lower the value of the parameter is—the partial Wilks’ lambda—the greater is the contribution of the element to the separation of fibres.

The discriminate functions were also obtained, which are combinations of the characteristic X-ray intensities of the elements used in the model.

D_1_ = 6.564 P − 1.314 Cl − 1.895 K + 2.229 Ca − 2.001 Cr − 2.063 Cu (1)

D_2_ = 1.012 P − 0.310 Cl + 1.491 K − 0.798 Ca − 1.474 Cr − 0.406 Cu (2)

**Figure 4:**
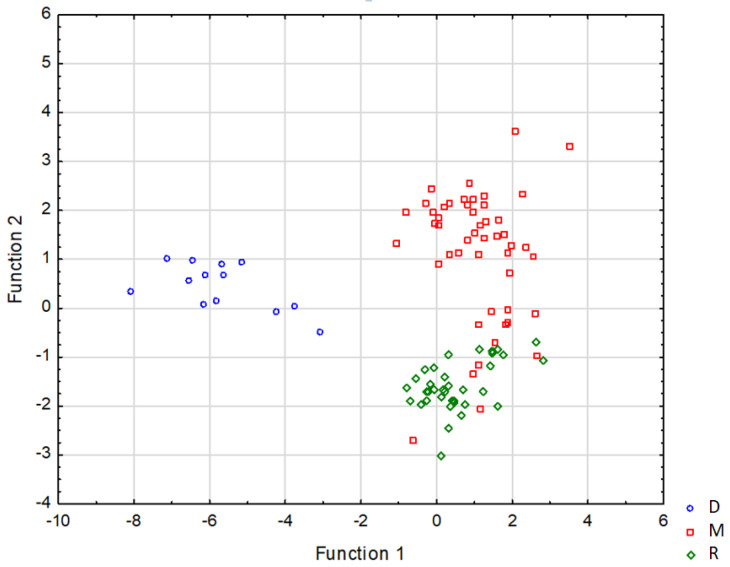
Graphic representation of the configuration of types of muscle fibres which are represented by points in the layout of discrimination variables.

For the first function, the greatest influence on the components was from P and Ca, while for the second function, the greatest influence was from K and Cr. The graphic representation of data distribution in the space of discriminate functions is presented in Figure 4. 

In order to verify the work of the created model, an inverse analysis was performed for the data used to develop the model. The results were compared with the original diagnosis. Overall, 93% of the fibres were matched to the category in which they were diagnosed (Table 2). From the fibres diagnosed as myopathy, six were assigned as the reference group. From the reference group, one was assigned as myopathy. Meanwhile, in the dystrophy group, all fibres showed the features of their assigned category. In the case of the fibre belonging to the reference group but assigned to the “myopathy” group, it was a fibre from a sample where the remaining fibres were unequivocally classified as myopathy. In the case of the six fibres diagnosed as myopathy and assigned as the reference, four came from one sample, of which the remaining fibres were ambiguously assigned to myopathy. Two of those fibres, however, belonged to a different sample in which they were the only fibres measured. They were unequivocally assigned to the reference group, which may indicate the initial disease processes which require further histopathological investigation.

In order to verify the correctness of the created model, an inverse analysis was performed for 30% of the randomly selected fibres, which were not involved in creating the model. The results were also compared with the original diagnosis (Table 3). For both the dystrophy and reference groups, the model achieved 100% correctness in assigning fibres to the appropriate group. In the case of myopathy, two fibres showed reference group characteristics, which gives us 87% correctness. Thus, the overall correctness of assigning fibres to the appropriate group in the model was 94%. Fibres assigned as references and diagnosed as myopathy were from the same sample as the four fibres involved in model building, also classified as reference. Here, first, it must be considered that when assigning fibres to one of the three given categories (D, M and R), all the measured fibres in the sample assigned to the category by diagnosis were taken into account. Not all the fibres that constitute the tissue in a given sample are in the same phase of pathological changes. Some can present advanced pathological changes, and others the initial ones. Additionally, the samples presented as a reference group came from patients suspected of having pathological changes, though histopathological diagnosis did not support clinical suspicions. However, the changes that have occurred in cells could be very slight and at a very early stage and thus could not be discernible in microscopic examination. The discovered changes may be more significant after a kind of preselection of the analysed fibres, which in turn may introduce a bias of some subjectivity. Nevertheless, such a non-random approach deserves to be tested in further analyses.

At this stage of study, factors such as medications, the possible presence of comorbidities, genetic factors, diet, etc., were not taken into account. Furthermore, it is necessary to keep in mind that samples of human origin can be affected by various factors, which unfortunately cannot be eliminated. This particularly relates to one of the more difficult aspects, which is the ability of patients to take medications and, especially, diet and eating habits. In this case, the biopsies were taken under the regime of short admission (hospitalisation) of the patient for a period of 1–2 days. Corticosteroid and other immunosuppressive therapy should be, if possible, discontinued prior to biopsies for a minimum of one month beforehand. However, medications critical to the patient’s health (e.g., cardiovascular) cannot be stopped. This situation in particular can occur for elderly patients with comorbidities. Therefore, it is difficult to speak about samples of human origin of a true control group. For this reason, fibres that were compared with others assigned as affected by disease changes were the reference group in which no fibres were assigned to the myopathy or dystrophy group.

## 3. Materials and Methods

**Figure 5:**
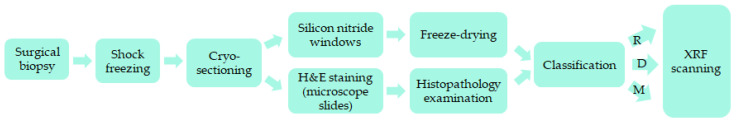
Schematic of the tissue sample preparation protocol. Abbreviations: D—dystrophies group; M—myopathy group; R—reference group.

The tissue material used in this study was obtained from surgical biopsies from patients with suspected muscle changes and then processed according to the standard neuropathological protocol used for muscle biopsy at the Department of Neuropathology at Jagiellonian University Medical College in Krakow (DN JUMC). The biopsy specimen being used for elemental micro-imaging was oriented on a holder to cut it in the plane strictly perpendicular to the long axis of the muscle fibres and then shock-frozen in isopentane previously cooled in liquid nitrogen. The muscle tissues prepared in this way were cut into slices of 8 µm thickness using a cryomicrotome at −20 °C. Samples intended for the elemental analysis were placed on silicon nitride windows (thickness: 200 nm; size: 2 × 2 mm^2^) (Norcada, Canada), while adjacent consecutive tissue slices were placed on microscope slides and stained with haematoxylin and eosin (H&E) for further histopathological examination. The samples prepared on the silicon nitride windows were freeze-dried at −80 °C. A schematic overview of the sample preparation protocol is presented in Figure 5. All the cases studied were diagnosed histopathologically in DN JUMC. The research was approved by the Jagiellonian University Medical College Ethics Committee (approval number: 1072.6120.249.2020).

For each sample the areas of interest in which the atrophic or hypertrophic fibres were present were selected. In the case of samples from the reference group, attention was paid to the places where the cell sizes were similar, and also to whether the fibres were in one so called fascicle or not.

**Figure 6:**
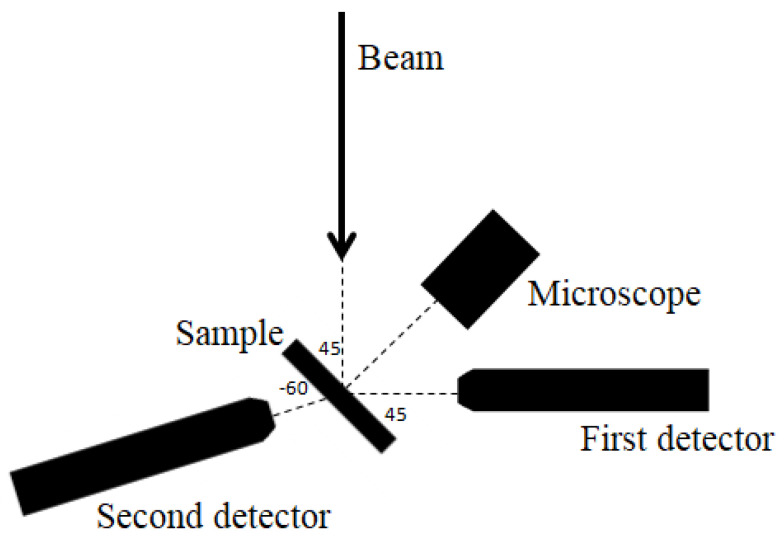
The scheme of the SR-XRF set-up used in the experiment.

The experiment was carried out at the I18 beamline at the DIAMOND Light Source. The beam size was shaped to the 5 × 5 µm^2^ size by the Kirkpatrick–Baez mirrors. The excitation energy was 13.5 keV. The recorded maps had different sizes depending on the number of measured fibres and their distribution. The scan step was 5 µm. The acquisition time of 4 s per pixel has been used. To detect the characteristic radiation two four-segment Vortex ME-4 SSD detectors were used—one in front of the sample at 45/45 deg geometry and one behind the sample at 45/−60 deg geometry (Figure 6). All measurements were performed in a helium atmosphere.

For the in SR-XRF investigation and according to the diagnosis, the samples were divided into three groups: dystrophy (D), myopathy (M) and a reference group (R). “Myopathy” here denotes muscle pathologies other than muscular dystrophy. The reference group consisted of samples taken from patients with suspected muscle disease where the pathomorphological investigation did not reveal evidence of pathology. All samples were collected from patients of both genders (male—m; female—f) and of different ages (41 ± 4 y), distributed as follows for all groups: dystrophies—*N* = 3: f (age: 56 y), m (age: 46 y) and m (age: 23 y); myopathies—*N* = 6: m (age: 30 y), m (age: 54 y), f (age: 32 y), f (age: 32 y), m (age: 32 y) and m (age: 30 y); reference group—*N* = 4: m (age: 50 y), m (age: 42 y), f (age: 35 y) and f (age: 75 y). Overall, 24 maps from 13 samples were used for measurements, from which areas of 135 fibres were separated. Of them, 17 were assigned as fibres affected by dystrophy, 64 as fibres affected by “myopathy” and 54 as a reference group. Due to the relatively small number of cases constituting the study group, no additional breakdown by age, gender, etc., was performed in the analysis.

**Figure 7:**
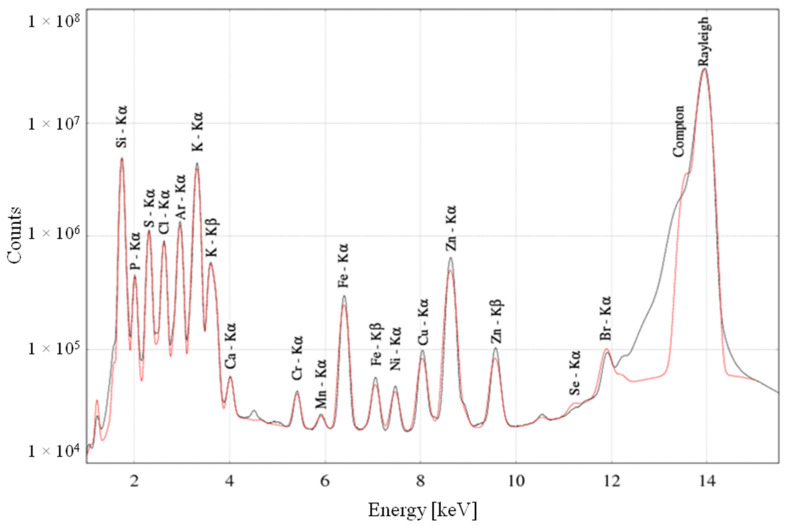
The spectrum of characteristic X-ray radiation of a selected fibre from the reference group. The measured spectrum is marked with the black line, and the red line shows the fit by the PyMca program.

**Figure 8:**
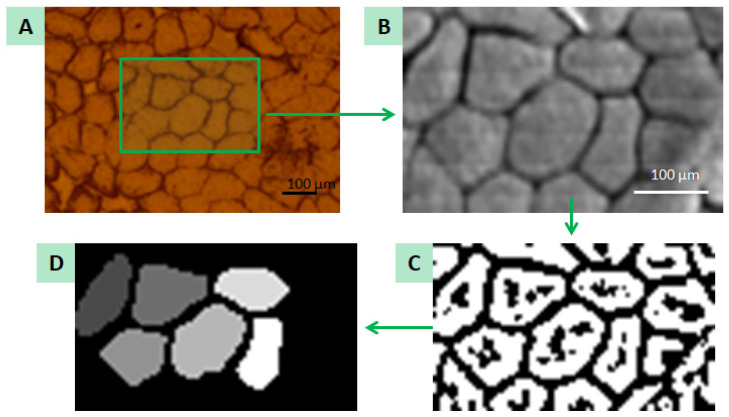
Graphical representation of steps needed to obtain the mask from an ROI image by binarisation: (**A**) a photo of the sample taken with an optical microscope with the marked area of measurement; (**B**) ROI image; (**C**) ROI image binarisation; (**D**) created mask.

Measurement data were saved in NeXus files. Then, the PyMca program [16] was used to obtain the ROI image—an image based on the integral over the entire spectrum—on the basis of which a binary image was created for each map. In order to binarise the image, the maps were pre-processed with Gaussian blur and then the Otsu method was applied using the Python environment [17]. The obtained images were processed into a mask to automate the allocation of pixels to a specific irregularly shaped fibre. Based on the binary image, each pixel was assigned a specific number depending on its location. The intercellular space—i.e., endomysium—was marked with the number 0; the fibres that were not completely measured were left with the number 1 and the pixels that made up the fibres that were completely measured were assigned the next natural numbers. The created mask was used to isolate the pixels that make up the irregularly shaped fibre. For this purpose, an original script in the Python environment was created. Using the NeXus files and the mask, the script was able to extract the appropriate spectra assigned to a specific fibre, sum them up and save the final spectrum in a separate ASCII file. The obtained spectra were fitted using the PyMca program in order to obtain information about the net area under the peaks of individual elements such as Br, Ca, Cl, Cr, Cu, Fe, K, Mn, P, S and Zn. Those values were normalised in two steps: to the Compton scatter peak [18] area values to minimise the influence of the sample thickness and density variations, and to the number of pixels corresponding to each fibre. An exemplary spectrum is shown in Figure 7. The data prepared in this way were then subjected to statistical analysis in the STATISTICA program. Figure 8 shows graphically the individual steps of creating the mask.

## 4. Conclusions

In summary, the above experiment, together with all the analyses performed, showed statistically significant differences in the elemental composition of the fibres classified as the reference group (R) and those representing the diseased tissue—dystrophy (D) and myopathy (M). Taking into account such statistical analyses as the Spearman correlation and multivariate discriminate analysis, the greatest impact on the differentiation of fibres can be attributed to elements such as Ca, K, Cr and P, which notably play an important role in the structure of fibres and nerve conduction. However, it must be taken into account that all fibres that have been measured for a relevant sample have been assigned to a given category by sample recognition; some of them could therefore be incorrectly assigned to a different category. This may be evidenced by the results obtained from the MDA. For this reason, some fibres may have been analysed at an advanced stage of the disease, and some only in its beginning. Further analyses after selecting the fibres, as well as taking into account factors such as diet, age, gender and co-morbidities, may show larger differences. Regarding our results, it seems that SR-XRF could be an important adjunct in the diagnosis of muscle disease, though it requires further studies.

## Figures and Tables

**Table 1:** Partial Wilks’ lambda value.

Element	Ca	K	P	Cu	Cl	Cr
Partial Wilks’ lambda	0.536	0.696	0.754	0.929	0.905	0.536

**Table 2:** Obtained classification matrix for matching fibres by multivariate discriminate analysis for 70% of randomly selected fibres. Abbreviations: D—dystrophies group; M—myopathy group; R—reference group.

	Group	%	Assignment			Total
			D	M	R	
Diagnosis	D	100%	13	0	0	13
	M	88%	0	43	6	49
	R	97%	0	1	37	38
	Total	93%	13	44	43	100

**Table 3:** Obtained classification matrix for matching fibres by multivariate discriminate analysis for 30% of randomly selected fibres, which were not involved in creating the model. Abbreviations: D—dystrophies group; M—myopathy group; R—reference group.

	Group	%	Assignment			Total
			D	M	R	
Diagnosis	D	100%	4	0	0	4
	M	87%	0	13	2	15
	R	100%	0	0	16	16
	Total	94%	4	13	18	35

## Data Availability

Data supporting this study cannot be made available due to ethical, reasons. The data includes the personal data of the patients.
